# Neutrophils with myeloid derived suppressor function deplete arginine and constrain T cell function in septic shock patients

**DOI:** 10.1186/cc14003

**Published:** 2014-08-01

**Authors:** Christabelle J Darcy, Gabriela Minigo, Kim A Piera, Joshua S Davis, Yvette R McNeil, Youwei Chen, Alicia D Volkheimer, J Brice Weinberg, Nicholas M Anstey, Tonia Woodberry

**Affiliations:** Global and Tropical Health Division, Menzies School of Health Research and Charles Darwin University, Casuarina NT 0811, P.O. Box 41096, Darwin, NT 0810 Australia; Infectious Diseases Department, Royal Darwin Hospital, Darwin, NT 0810 Australia; Division of Hematology-Oncology, Duke University and Veterans Affairs Medical Centers, Durham, NC 27705 USA

## Abstract

**Introduction:**

Impaired T cell function in sepsis is associated with poor outcome, but the mechanisms are unclear. In cancer, arginase-expressing myeloid derived suppressor cells (MDSCs) deplete arginine, impair T cell receptor CD3 zeta-chain expression and T cell function and are linked to poor clinical outcome, but their role during acute human infectious disease and in particular sepsis remains unknown. Hypoarginemia is prevalent in sepsis. This study aimed to determine whether neutrophils that co-purify with PBMC express arginase, and if arginine depletion constrains T cell CD3 zeta-chain expression and function in human sepsis.

**Methods:**

Using flow cytometry, cell culture, HPLC, arginase activity and mRNA detection, our study examined whether neutrophils, with reduced buoyant density isolated in the Ficoll interface, metabolise L-arginine and suppress T cell proliferation in sepsis. A total of 35 sepsis patients (23 with septic shock) and 12 hospital controls in a tertiary referral hospital in tropical Australia were evaluated.

**Results:**

Only sepsis patients had interphase neutrophils, neutrophils co-purifying with mononuclear cells (≤1.077 specific gravity). The percentage of interphase neutrophils in sepsis was proportional to sepsis severity and correlated with plasma IL-6 concentrations. *Ex vivo,* sepsis-derived interphase neutrophils expressed arginase, metabolised culture L-arginine and suppressed T cell proliferation and CD3 zeta-chain expression. *In vivo*, in septic shock there was a longitudinal inverse association between interphase neutrophil number and CD3 zeta-chain expression. Depletion or inhibition of interphase neutrophils *in vitro* restored zeta-chain expression and T cell function.

**Conclusions:**

For the first time during an acute human infection, interphase neutrophils that express arginase were found to circulate in sepsis, in proportion to disease severity. These neutrophil-MDSCs impair T cell CD3 zeta-chain expression and T cell function via L-arginine metabolism, and likely contribute to the T cell dysfunction seen in sepsis. Modulation of neutrophil-MDSC or their downstream effects warrant consideration as targets for novel adjunctive therapies in sepsis.

**Electronic supplementary material:**

The online version of this article (doi:10.1186/cc14003) contains supplementary material, which is available to authorized users.

## Introduction

Sepsis is a systemic inflammatory response to infection [1]. Despite improvements in its management, septic shock has a mortality rate of 30 to 50% [2–4] and is a leading cause of death in ICUs [2].

Although sepsis patients have high levels of inflammatory mediators, some components of their immune system are strongly suppressed [5, 6], and sepsis has been described as an immunosuppressive disorder or a state of immunoparalysis [7, 8]. Clinical trials demonstrate that anti-inflammatory and immunosuppressive therapies may be harmful in sepsis and septic shock [9, 10]. *In vivo* evidence of T cell dysfunction in sepsis is demonstrated by impaired delayed-type hypersensitivity [11] and cytomegalovirus and herpes simplex virus re-activation [12, 13]. This is supported *ex vivo* by impaired T cell proliferation, cytokine production [14], and lymphocyte apoptosis [15]. Loss of T cell function is associated with sepsis mortality [14, 16], other poor outcomes [15] and decreased resistance to secondary infections [17]. The mechanisms of T cell suppression in sepsis remain incompletely understood.

Sepsis patients have decreased plasma concentrations of L-arginine [18], a conditionally essential amino acid critical for immune function and for surface expression of a fully functional T cell receptor (TCR) [19]. The TCR trans-membrane molecule consists of an antigen-specific αβ heterodimer receptor coupled to invariant γδϵ and ζ homodimer chains that mediate signal transduction - enabling T cell proliferation and cytokine secretion. *In vitro* L-arginine depletion impairs T cell zeta-chain expression and cell proliferation, which both recover when L-arginine is restored [19, 20]. Arginase or arginase-producing cells also impair T cell zeta-chain expression through local depletion of L-arginine [21, 22]. Our previous characterisation of reduced L-arginine levels in sepsis patients [18] led to the hypothesis that T cell zeta-chain downregulation contributes to T cell dysfunction in sepsis.

Myeloid-derived suppressor cells (MDSC) are a heterogenous group of cells which can downregulate T cell receptor zeta-chain expression. MDSC suppress T cell activation and proliferation and have been described in cancer patients [23], trauma patients [24], healthy volunteers systemically challenged with endotoxin [25], mouse models of sepsis [26] and other murine infections [27, 28]. In human peripheral blood two major subpopulations of MDSC are described; granulocytic and monocytic. Monocytic MDSC express CD14 and exert suppression via arginase, iNOS and suppressive cytokines [29]. Granulocytic or neutrophil-MDSC express CD15 and may suppress via the production of arginase or reactive oxygen species [29]. Activated neutrophil MDSC have been shown to co-purify with peripheral blood mononuclear cells (PBMC) after density gradient separation [24, 30, 31]. As immature neutrophils have been reported in PBMC from three patients with sepsis [32], we hypothesised that neutrophils co-purifying with PBMC in sepsis are activated MDSC which suppress T cells via arginase.

Here we report that sepsis patients have impaired T cell zeta-chain expression and patients with shock have significantly more neutrophils co-purifying with PBMC compared to sepsis patients without shock. These low density neutrophils suppress T cell proliferation and *in vitro* depletion restores T cell zeta-chain expression and T cell proliferative capacity. Consequently, these cells can be considered neutrophil-MDSC. These data provide a mechanism for T cell dysfunction in adults with severe sepsis and suggest the potential for adjunctive therapies to restore T cell function and improve outcome.

## Materials and methods

### Cohort

Sepsis patients had suspected or confirmed infection, the presence of two or more criteria for the systemic inflammatory response syndrome (SIRS) within the last 4 hours [1], and were classified as having septic shock, or sepsis without shock. Septic shock was defined at the time of enrolment as systolic blood pressure <90 mmHg or a reduction ≥40 mmHg from baseline despite adequate fluid resuscitation, or the need for vasopressors to maintain these targets [1]. Sepsis severity was estimated using the acute physiology and chronic health evaluation (APACHE) II score from the first 24 hours of admission and daily modified sequential organ failure assessment (SOFA) score. Patients were enroled within 24 hours of ICU admission or within 36 hours of ward admission. Control subjects were recruited from hospital patients who had not met SIRS criteria within the last 30 days and who had no clinical or laboratory evidence of inflammation or infection. Written informed consent was obtained from all participants or next of kin. The study was approved by the Human Research Ethics Committee of Menzies School of Health Research and the Department of Health and Community Services.

### Blood collection, sample preparation and lymphocyte counts

Venous blood was collected in lithium heparin tubes at enrolment (day 0), days 2 to 4, and day 7 until discharge from the hospital or death. Whole-blood differential white cell counts were measured by Coulter Counter. Plasma was separated within 30 minutes of collection and stored at −80°C. To exclude *ex vivo* neutrophil density changes, cells were separated within 2 hours of collection using Ficoll-Hypaque™ Plus (GE Healthcare Biosciences, Uppsala, Sweden) density gradient. Interphase cells (those at the 1.088 specific gravity interphase), including neutrophils and/or PBMC, were either stained fresh or cryopreserved in liquid nitrogen in 90% heat-inactivated foetal calf serum (GIBCO, Invitrogen, USA) and 10% dimethyl sulfoxide (Sigma, USA). Neutrophils collected from the interface of Ficoll, that is, co-located with PBMC in the interphase layer were termed interphase neutrophils. In a subset of samples, polymorphonuclear neutrophils (PMN) were collected from beneath the Ficoll-Hypaque™ Plus layer (bottom fraction).

### Leukocyte/cell preparation for microscopy

Cells separated by density gradient into the interphase and bottom fractions were prepared for microscopy by cytospin. Samples were prepared with a cell suspension of 50,000 cells in 100 μL of plasma. Centrifugation was performed in a Shandon cytospin 4 (Thermo Fisher Scientific, Australia) for 8 minutes at 800 revolutions per minute (rpm). Preparations were fixed with Quick Dip Fixative (Fronine Australia) and stained with Quick Dip (Fronine). Manual leukocyte differentiation was performed using a Zeiss microscope.

### Flow cytometric evaluation

All longitudinal samples cryopreserved in liquid nitrogen were thawed simultaneously and tested in a single experiment. The proportion of interphase neutrophils in total interphase cells was calculated in 23 out of 24 sepsis patients and all control patients. For cryopreserved samples, media with 50 units/mL benzonase nuclease (Novagen, Denmark) was used in thawing to reduce cell clumping, and the interphase cells were immediately stained and analysed. Freshly-isolated cells were examined to confirm quantification of neutrophils in fresh versus thawed samples and to validate phenotypic and functional analyses.

Antibodies to CD3, CD16, CD56, CD11b, CD15, CD33, CD49d, CD54 and CD62L were sourced from Biolegend (CA, USA); CD4, CD8, CD66b and CD14 from BD Biosciences (Pharmingen, CA, USA); and CD155 from eBioscience (CA, USA). CD247 (Beckman Coulter, Immunotech, USA) was used to measure CD3 zeta-chain expression in cells surface stained, fixed in 0.25% paraformaldehyde (Sigma) [33] and permeabilised with 100 μg/mL digitonin (Cayman chemical company, Michigan, USA) while kept on ice for 10 minutes. To control for inter-experimental variation in CD3 zeta-chain mean fluorescence, cells from a single donation, from a healthy donor, were cryopreserved in aliquots and one aliquot was thawed with every experiment to establish a normalization factor. All cryopreserved cells and a proportion of freshly isolated cells were read on a FACSCalibur flow cytometer (Becton Dickinson Immunocytometry Systems, MA, USA) and analysed using Flow Jo software (Tree Star, Oregon, USA). Freshly-isolated cells and some cryopreserved cells were analysed later using a Gallios flow cytometer (Beckman coulter) and Kaluza 1.2 for data analysis.

### Isolation of interphase neutrophils and T cell proliferation assays

Interphase neutrophils were enriched from total interphase cells by labeling with CD66b FITC (BD Biosceinces) followed by anti-fluorescein isothiocyanate (FITC) magnetic bead selection (MACS, Miltenyi Biotech, USA) according to manufacturer’s instructions. Proliferation assays were arranged in 96- or 48-well plates using a minimum of 200,000 cells/well of either total interphase (including interphase neutrophils) or after depletion of CD66b + interphase neutrophils. For some experiments isolated interphase neutrophils or PMN were added back to cultures after CD66b-depletion. Proliferation was determined by labeling cells with 100 μM carboxyfluorescein diacetate succinimidyl ester (CFSE, Invitrogen) and stimulating with immobilized anti-CD3 (1 μg/mL OKT-3; Biolegend) and soluble anti-CD28 (0.1 μg/mL CD28.2, Biolegend) antibodies. Proliferation was measured 3 to 4 days later by flow cytometry following surface staining of T cells. Cell division was confirmed by stimulating cells and on day 4 staining with Ki67 (BD Biosciences). Culture experiments used custom-formulated Advanced RPMI 1640 (Formula number 07-5074EA, GIBCO, Invitrogen) with L-arginine at 150 μM. The selective, competitive and high-affinity inhibitor of arginase N-Hydroxy-nor-L-arginine, diacetate salt (nor-NOHA) (Calbiochem, CA, USA) was used at a final concentration of 50 μM in culture as indicated. The peroxynitirite scavenger uric acid (Sigma, USA), was added to culture at a final concentration of 500 μM.

### mRNA detection of arginase I and II

We prepared total RNA from cell pellets using Qiagen RNeasy Mini Kit (catalogue number 74104 Qiagen, USA) following the manufacturer’s protocol. cDNA was prepared using the High Capacity cDNA Reverse Transcription Kit (Applied Biosystems catalogue number 436881 USA) following the manufacturer’s protocol. Quantitative PCR for arginase 1 and arginase 2 (and the control genes *GAPDH* and *HPRT1*) was performed using TaqMan master mix and probes from Applied Biosystems and following manufacturer’s protocol. Fold changes were calculated using the ΔΔ^ct^ method. The primers and probes were designed using Universal Probe Library Assay Design Center on the Roche website [34]. The probes were purchased from Roche Universal Probe Library, and the primers were purchased from Integrated DNA Technologies (IDT) (Additional file 1: Table S1).

### Plasma L-arginine and arginase activity

Plasma L-arginine concentrations were measured by high pressure liquid chromatography (HPLC; Shimadzu, Kyoto, Japan) with UV (250 nm) and fluorescence (excitation 250 nm, emission 395 nm) detection [35]. Plasma arginase activity was measured using a radiometric assay, as previously described, and reported as micromole/milliliter/hour [36].

### Plasma cytokine measurements

Concentrations of plasma IFN-γ, IL6 and IL10 were determined using a cytometric bead array (Human Th1/Th2 Cytokine Kit II, BD Biosciences Pharmingen, CA, USA) and analysed using FCAP array version 1.0.1 (Soft Flow Hungary for Becton Dickinson Biosciences). The lower limits of detection (LLD) of the assay were 2.5 pg/mL for IFN-γ and 10 pg/mL for IL6 and IL10.

Concentrations of plasma vascular endothelial growth factor (VEGF) were determined using the R&D human VEGF Quantikine® ELISA in accordance with the manufacturer’s instructions. The lower limit of detection was 31 pg/mL. Values below the lower limits of detection (LLD) were assigned a value halfway between zero and the LLD for statistical analysis.

### Statistical methods

Groups for analysis were septic shock, sepsis without shock and hospital controls. Continuous non-normal variables were compared using the Mann-Whitney test. Correlation was examined using Pearson’s test. Linear mixed-effects models were used to examine longitudinal correlation. A two-sided *P*-value <0.05 was considered significant. Analyses were performed using Stata version 10.0 (Stata Corp TX, USA) and Prism version 5.01 (GraphPad Software).

## Results

### Participants

We initially studied cryopreserved PBMC only from 24 patients with sepsis and 12 hospital controls (Table 1), enroled in a previously reported longitudinal study of endothelial function in sepsis [37], that were representative of the entire cohort in terms of age, gender, ethnicity and disease severity. Fresh and cryopreserved samples from an additional 11 septic shock patients (Table 2) were used for cell separation studies at a later time.

**Table 1:** **Characteristics of sepsis patients and hospital controls**

	Sepsis with shock	Sepsis no shock	Hospital controls	Sepsis shock versus no shock	All sepsis versus control
Subjects, n	12	12	12		
Age, years	52 (45 to 57)	45 (39 to 55)	49 (40 to 56)	ns	ns
Male, n (%)	7 (58%)	6 (50%)	8 (67%)	ns	ns
ATSI, n (%)	10 (83%)	7 (58%)	8 (67%)	ns	ns
APACHE II score, day 0	20 (29 to 23)	8 (4 to 14)		<0.0001	N/A
SOFA score, day 0	10 (4 to 10)	1 (0 to 2)		<0.0001	N/A
Plasma L-arginine, μM	39 (25 to 53)	40 (21 to 48)	74 (65 to 88)	ns	<0.0001
Plasma IL-6, pg/mL	1433 (400 to 4290)	82 (42 to 302)	5 (5 to 5)	<0.0001	<0.0001
Plasma IL-10 (pg/mL), median (range)	65 (5 to 9525)	5 (5 to 72)	5 (5)	0.001	0.004
Plasma VEGF, pg/mL	89 (16 to 115)	79 (62 to 138)	51 (32 to 71)	ns	0.03
Plasma arginase, μmol/mL/hr	0.18 (0.1 to 0.23)*	0.19 (0.12 to 0.26)	0.14 (0.09 to 0.16)	ns	ns
Interphase-neutrophils (%)	19.2 (4.4 to 29.5)	2.7 (1.5 to 6.1)	1.5 (0 to 2.0)	0.02	0.001
Neutrophil × 10^3^/μL	13.1 (7.2 to 19.4)	14.2 (11.4 to 16.6)	6 (4.0 to 9.6)	ns	0.02
Imm. granulocyte × 10^3^/μL, median (range)	0.4 (0 to 11.8)	0 (0 to 7.6)	0 (0 to 0)	0.05	N/A
Monocyte × 10^3^/μL	0.45 (0.1 to 1.2)	0.65 (0.4 to 1)	0.55 (0.5 to 0.7)	ns	ns
Lymphocyte × 10^3^/μL	1.2 (0.5 to 2.1)	1.2 (0.8 to 1.6)	2.2 (1.5 to 2.4)	ns	ns
Causative organism, n (%)					
None cultured	5 (42%)	9 (75%)			
Gram-positive bacteria	4 (33%)	2 (17%)			
Gram-negative bacteria	3 (25%)	1 (8%)			

**Figure 1:**
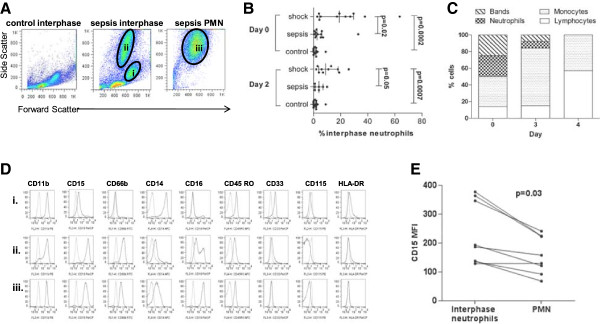
**Phenotypic characterisation of interphase neutrophils in sepsis patients. (A)** Flow cytometric evaluation of interphase cells from a representative healthy control and interphase cells and PMN from a representative septic shock patient. Gated regions illustrate: (i) monocytes, (ii) interphase neutrophils and (iii) PMN. The proportion of interphase neutrophils was determined using flow cytometry by gating region ii, and expressed as a percentage of all viable cells. **(B)** The proportion of interphase neutrophils collected following gradient separation of blood from patients with septic shock (n = 12), sepsis without shock (n = 12) and hospital controls (n = 12) on day 0 (enrolment) and day 2. Horizontal lines show the median and interquartile range. **(C)** Microscopic identification of interphase cells in longitudinal samples from a representative septic shock patient. **(D)** Fluorescence-activated cell sorting (FACS) Calibur flow cytometric phenotyping of (i) monocytes, (ii) interphase neutrophils and (iii) PMN from a representative septic shock patient. The first peak represents background or negative staining. **(E)** Gallios median fluorescence intensity (MFI) of CD15 expressed on paired non-cryopreserved interphase neutrophil and polymorphonuclear neutrophils (PMN) from four septic shock patients (three with a longitudinal day-2 or day-3 sample). *P*-values were derived using a non-parametric analysis of the seven paired interphase neutrophil and PMN data points.

Values show the median (interquartile range) unless stated otherwise. *n = 11, Interphase neutrophil (%) indicates the proportion of neutrophils in the total interphase layer (gated per Figure 1A). ATSI, Aboriginal or Torres Strait Islander; APACHE, acute physiology and chronic health evaluation; SOFA, sequential organ failure assessment; VEGF, vascular endothelial growth factor; N/A, not assessed; ns, not significant.

**Table 2:** **Characteristics of additional sepsis patients enroled for cell separation and functional studies**

	Sepsis with shock
Subjects, n	11
Age, years	50 (37 to 70)
Male, n (%)	11 (100%)
ATSI, n (%)	6 (55%)
APACHE II score, day 0	15 (13 to 20)
SOFA score, day 0	7 (6 to 8)
Causative organism, n (%)	
None cultured	4 (36%)
Gram-positive bacteria	4 (36%)
Gram-negative bacteria	3 (27%)

ATSI, Aboriginal or Torres Strait Islander; APACHE, acute physiology and chronic health evaluation; SOFA, sequential organ failure assessment.

### Neutrophils co-purify with PBMC in septic shock patients

Interphase cells from septic shock patients collected after density gradient separation contained atypical neutrophils with reduced buoyant density (≤1.077 specific gravity), referred to as interphase neutrophils. Flow cytometry revealed a similar forward and side scatter profile (representing size and granularity) for both the interphase neutrophils and the polymorphonuclear neutrophils (PMN) recovered from below the Ficoll-Hypaque layer (Figure 1A). Cryopreserved interphase cells from 12 septic shock patients, 12 non-shock sepsis patients, and 12 hospital controls (Table 1) were assessed for the presence of interphase neutrophils. Only sepsis patients had detectable interphase neutrophils (≥10% of all viable cells). Septic shock patients had significantly more interphase neutrophils compared to sepsis patients without shock, both on the day of enrolment (*P* = 0.02 Figure 1B) and on day 2 (*P* = 0.05 Figure 1B). The interphase neutrophils in septic shock patients were a mix of bands (immature) and segmented (mature) cells by microscopy and were found at multiple, but not all, longitudinal time points (Figure 1C and Additional file 2: Figure S1). Detailed phenotypic characterisation of interphase neutrophils in fresh and frozen samples revealed high expression of the granulocyte markers CD66b (a glycosylphosphatidylinositol linked protein), CD15 (a carbohydrate structure), and CD11b (an integrin expressed by monocytes and granulocytes that is upregulated on neutrophils after activation) (Figure 1D). Interphase neutrophils also expressed CD45RO (leukocyte common antigen), CD49d (integrin) and intermediate levels of CD16 (the Fcγ III receptor) (Figure 1D and Additional file 3: Figure S2). The interphase neutrophils were negative or low for CD33 (a transmembrane glycoprotein expressed on monocytes and myeloid progenitors), CD62L (L-selectin) or CD54 (ICAM-1), the major histocompatability complex class II antigen HLA DR and the monocyte markers CD14 and CD115 (colony stimulating factor -1 receptor) (Figure 1D and Additional file 3: Figure S2). In only the fresh (non-cryopreserved) samples, the more buoyant interphase neutrophils were compared to PMN and found to express significantly more CD15, while CD16 (total or the high population) was similarly expressed between both (Figure 1E). The lack of CD33 expression distinguishes these cells from most human neutrophil-MDSC previously described [29] and intermediate to high CD16 expression and the lack of CD62L or CD54 distinguishes these cells from those previously described to be induced by endotoxin challenge by Pillay [25]. In total, the phenotype of the interphase neutrophil fits that of a mature neutrophil [38], and in shock patients the percentage of interphase neutrophils correlated with the mature PMN count derived from an automated cell counter.

### Numbers of interphase neutrophils correlate with low CD3 zeta-chain expression

Cryopreserved cells collected longitudinally from patients with septic shock, non-shock sepsis, and hospital controls were tested to measure associations between sepsis disease severity, T cell zeta-chain expression, and the percentage of interphase neutrophils. To allow the direct comparison of longitudinal sepsis and control samples, cells were cryopreserved and all samples from each patient run on the same day with a zeta control that was run at every experiment. Only cryopreserved cells were evaluated for T cell zeta-chain allowing the direct comparison of longitudinal sepsis and control samples within experiments.

**Figure 2:**
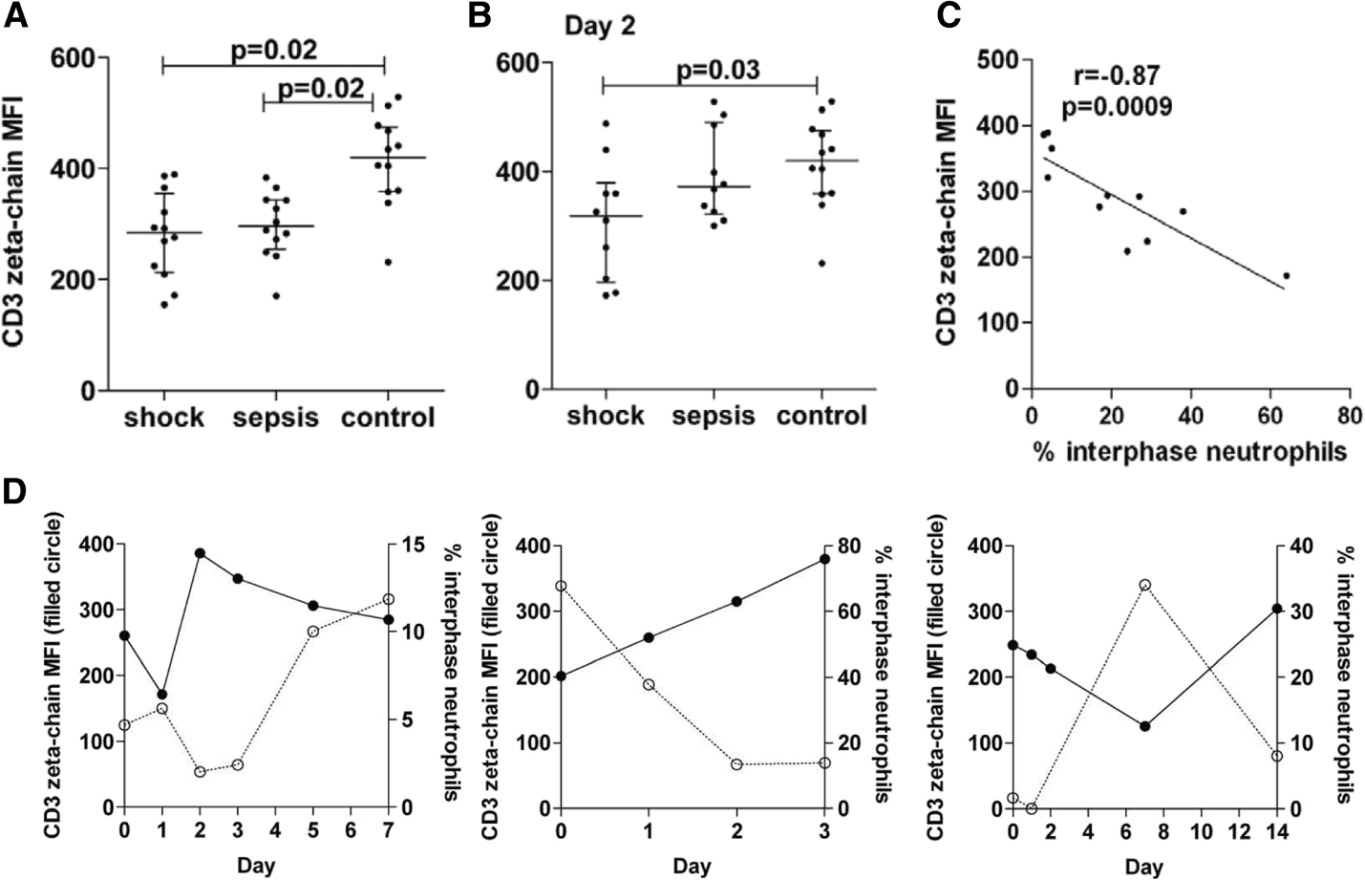
**Inverse association between T cell zeta-chain expression and interphase neutrophil number. (A)** Gated T cell CD3 zeta-chain expression *ex vivo* in patients with septic shock (n = 12), sepsis without shock (n = 12), and hospital controls (n = 12) on day 0 (enrolment). **(B)** On day 2, zeta-chain expression remained low in septic shock patients but recovered for non-shocked patients. Horizontal lines show the median and interquartile range. **(C)** Inverse association between the proportion of *ex vivo* interphase neutrophils and T cell zeta-chain expression in patients with septic shock (n = 11) on day 0. **(D)** Longitudinal *ex vivo* CD3 zeta-chain expression (left axis, filled circles) and the percentage of interphase neutrophils (right axis, empty circles) in three representative patients with septic shock. This longitudinal inverse association was statistically significant in a mixed effects model (*P* = 0.02).

On admission, septic shock and non-shock patients had significantly reduced T cell zeta-chain expression (Figure 2A). However, by day 2, non-shock patients had recovered T cell zeta-chain expression, while septic shock patients with interphase neutrophils had not (Figure 2B). In six septic shock patients with a day-7 sample there was no significant recovery in T cell zeta-chain expression, suggesting continued immunosuppression for at least one week. In the septic shock patients, there was a strong inverse correlation between the number of interphase neutrophils and expression of the T cell zeta-chain, both on admission (day 0 *r* = −0.87, *P* = 0.0009; Figure 2C) and day 2 (*r* = −0.85, *P* = 0.004). The inverse association between CD3 zeta-chain expression and the interphase neutrophil number identified in cross-sectional samples was particularly evident in longitudinal *ex vivo* samples from patients with septic shock (Figure 2D). The *ex vivo* longitudinal inverse association was statistically significant in a mixed effects model (*P* = 0.02), suggesting that interphase neutrophils mediate T cell suppression via downregulation of the CD3 zeta-chain.

In all sepsis patients, there was a positive correlation between the interphase neutrophil number and plasma concentrations of IL-6 on the day of admission (day 0, *r* = 0.62, *P* = 0.002). In contrast, there was no association between plasma concentrations of VEGF on the day of admission and interphase neutrophil number in any of the sepsis patients (*r* = −0.04, *P* = 0.84).

**Figure 3:**
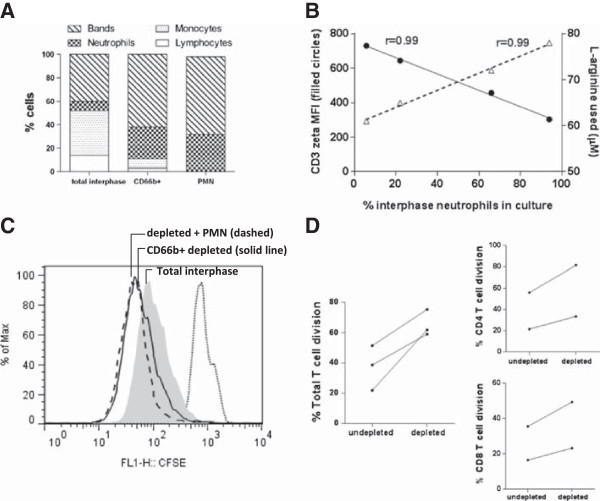
**Functional characterisation of interphase neutrophils in sepsis. (A)** Microscopic evaluation of total interphase cells, enriched CD66b + interphase neutrophils (CD66b+), and polymorphonuclear neutrophils (PMN) (from the bottom fraction) from a single representative septic shock patient. **(B)** Total interphase cells were depleted of interphase neutrophils (leaving PBMC), and the enriched CD66b + interphase neutorophils were returned at 6%, 22%, 66% and 94% to PBMC. After 48 hours of culture, there was an inverse association between gated T cell zeta-chain expression (left axis, filled circles) and culture L-arginine concentrations (right axis, empty triangles). **(C)** Proliferation of interphase cells, gated only on T cells, from a representative severe sepsis patient (grey fill) and enhanced proliferation (left shift) after depletion of interphase neutrophils (black solid line). Plots represent a minimum of 50,000 T cells per condition. Enhanced proliferation remained with addition of PMN (from bottom fraction, black dashed line) at a ratio of 1:2. Control, unstimulated interphase cells are designated by the dotted line. **(D)** Lymphocyte and CD4 T cell and CD8 T cell division of interphase cells from severe sepsis patients before and after CD66b + depletion.

*In vitro* culture experiments examined the direct effect of interphase neutrophils on T cell CD3 zeta-chain expression. We used FITC-labelled antibodies to CD66b and magnetic beads to isolate and enrich interphase neutrophils (Figure 3A). In a different individual, isolated CD66b cells (which contained 94% CD66b + interphase neutrophils with 1.4% lymphocytes and 1.9% monocytes) were then returned to CD66-depleted interphase cells at increasing concentrations and cultured. Culture experiments demonstrated an inverse association between CD3 zeta-chain expression and the CD66b + interphase neutrophils (Figure 3B). Culture data confirmed the inverse associations observed in *ex vivo* cross-sectional (Figure 2C) and longitudinal (Figure 2D) samples, and established that only the CD66b + interphase cells were responsible. In culture supernatant, L-arginine consumption increased with increasing numbers of interphase neutrophils (Figure 3B).

### Interphase neutrophils suppress T cell function in septic shock

T cell proliferation experiments sought to determine whether interphase neutrophils compromised T cell division. CD66b + interphase neutrophils were depleted from interphase cells using magnetic beads, and T cell proliferation was tested following CD3 and CD28 stimulation, either in the presence or absence of the CD66b + interphase neutrophils. Presence of CD66b + interphase neutrophils suppressed T cell proliferation (Figure 3C and Additional file 4: Figure S3), with both CD4+ and CD8+ T cell division compromised (Figure 3D). The interphase neutrophils were able to suppress the highly stimulatory T cell responses elicited by plate-bound anti-CD3 plus soluble anti-CD28. Suppression of proliferation was mediated by only the interphase neutrophils that co-purify with the PBMC and not the PMN (Figure 3C). While the number of interphase neutrohpils appeared to determine the inhibition of T cell division, heterogeneity was observed in the timing (day) of suppression within individuals.

### Interphase neutrophil L-arginine metabolism mediates T cell suppression

**Figure 4:**
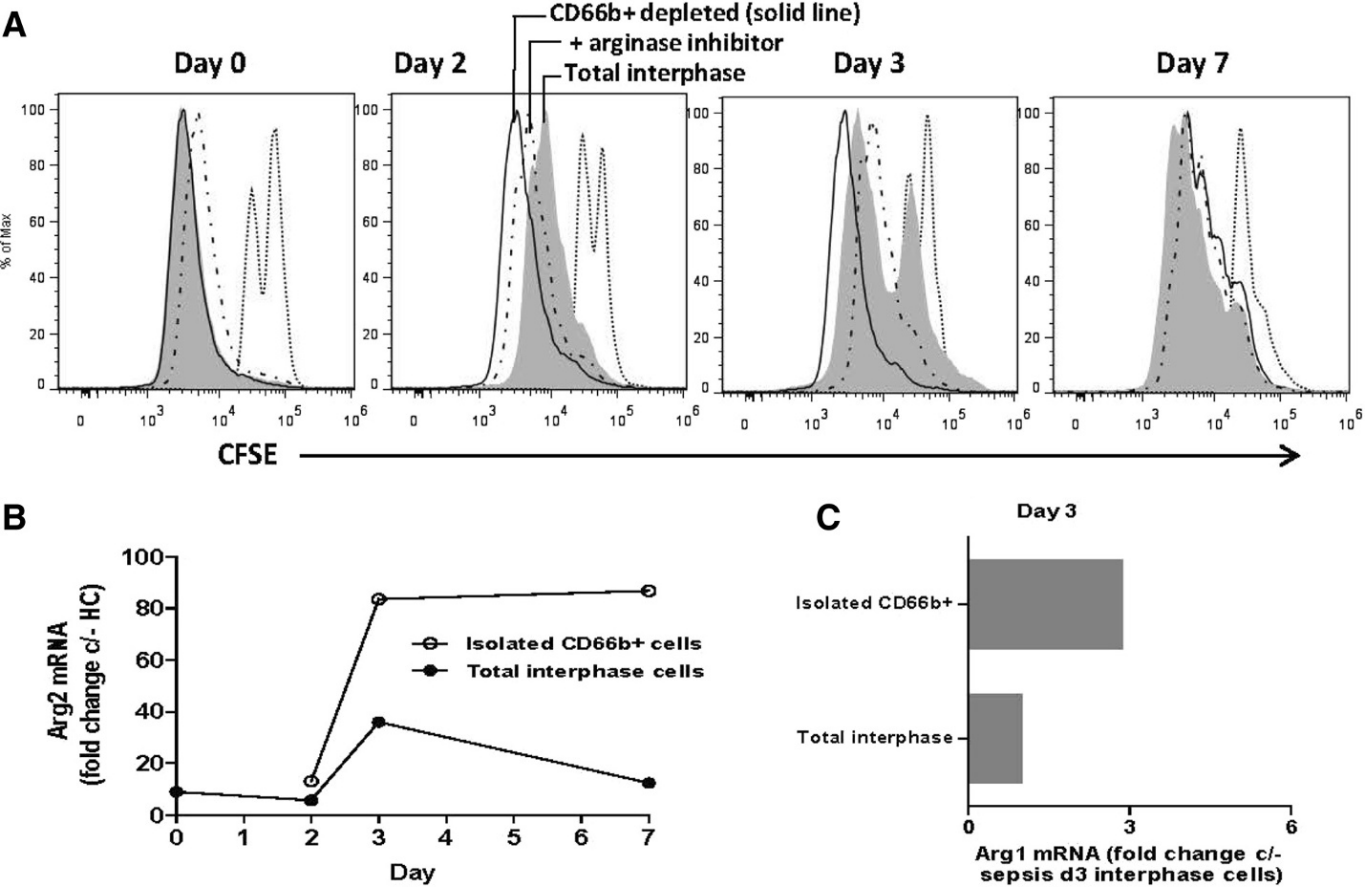
**Arginase expression by interphase neutrophils. (A)** T cell proliferation in longitudinal (day 0, 2, 3, and 7) samples from a representative severe sepsis patient. At each time point, T cell responses were measured in total interphase cells (grey fill), with addition of an arginase inhibitor (black hashed line), and after CD66b + interphase neutrophil depletion (black solid line). Only on day 2 and 3, removal of interphase neutrophils- enhanced proliferation. On day 2, addition of the arginase inhibitor also increased proliferation. No increased proliferation was observed on day 0 and 7 following depletion of interphase neutrophils, suggesting the interphase neutrophils were inactive. Control interphase cells in the absence of stimulation are designated by the dotted line. **(B)** In the same severe sepsis patient, mRNA was extracted from total interphase cells and the enriched interphase neutrophils at all longitudinal (day 0, 2, 3, and 7) time points. The line graph shows the fold change in arginase 2 mRNA expression in total cells (filled circles) and the interphase neutrophils (open circles) relative to interphase cells (PBMC) from a healthy control. **(C)** In the same severe sepsis patient, arginase 1 mRNA expression was evaluated at each time point in total interphase cells and the enriched interphase neutrophils. Arginase 1 expression was only detected on day 3 in both total interphase cells and the interphase neutrophils.

We hypothesised that interphase neutrophils suppress T cell function and CD3 zeta-chain expression via L-arginine depletion. In proliferation assays, addition of the arginase inhibitor nor-NOHA restored T cell division (Figure 4A). Evidence for the specificity of the inhibition of interphase neutrophil arginase included the lack of inhibition by nor-NOHA of T cell proliferation in cells from control patients (none of which had interphase neutrophils), and the inability of the peroxynitrite scavenger uric acid to inhibit T cell proliferation in cells from sepsis patients. In addition, in longitudinal sepsis samples, nor-NOHA increased T cell proliferation only when interphase neutrophil function was demonstrated (that is, only on day 2, when depletion of the neutrophil-MDSC restored proliferation, Figure 4A day 2). No increased proliferation was observed on day 0 and 7 following depletion of interphase neutrophils, suggesting the interphase neutrophils were inactive.

Arginase was not detected in the culture supernatant of total interphase cells, or the enriched CD66b + interphase neutrophils after 24, 48 or 96 hours incubation, suggesting there was no cell lysis or arginase secretion. Nor was there supernatant evidence that interphase neutrophils from sepsis patients produced cytokines (IL-2, IL-4, IL-6, IL-10, TNF or IFNγ) when cultured *in vitro* (unstimulated total cells or enriched CD66b + interphase neutrophils).

Arginase expression by the sepsis interphase neutrophil was confirmed by PCR. At all longitudinal time points (in two individuals), interphase cells expressed arginase 2. Arginase 2 mRNA levels were at least 10-fold higher in septic shock cells than healthy controls (Figure 4B). Data from isolated interphase neutrophils showed these cells alone were predominantly responsible for the arginase 2 mRNA expression detected in the total interphase cells (Figure 4B). Arginase 1 mRNA was also detected in total interphase cells and the isolated interphase neutrophils, coinciding with the day of peak interphase neutrophil activity (Figure 4C).

## Discussion

This study is the first description of interphase neutrophils metabolising arginine and constraining T cell function during an acute human infectious disease. Septic shock patients have increased numbers of circulating interphase neutrophils that downregulate expression of T cell zeta-chain and suppress T cell function. Arginase-mediated metabolism of L-arginine by these cells suppresses T cell proliferation; hence, these low-density neutrophils are MDSC. Sepsis patients with higher numbers of circulating interphase neutrophil MDSC had more severe disease, higher concentrations of plasma IL-6 and slower recovery of T cell zeta-chain expression. The slower recovery of T cell zeta-chain expression in septic shock patients is consistent with reports that patients with more severe sepsis have longer durations of T cell dysfunction [5, 14]. Slower recovery of T cell function appears at least partially attributable to interphase neutrophil-MDSC, suggesting these cells contribute to T cell dysfunction linked to greater risk of secondary bacterial infections [17] and mortality in sepsis [14, 16].

MDSC are evident in cancer and inflammatory bowel disease [29], chronic hepatitis C [39], HIV [40], and cystic fibrosis patients with chronic *P. aeruginosa* infection [41], but there are no reports of them in any acute human infectious disease, except in a few cases of convalescent influenza [42]. MDSC have been described in a mouse cecal ligation and puncture model of sepsis where MDSC appear to improve survival [43]. In contrast, our results from humans with sepsis suggest that excessive numbers of circulating interphase neutrophil MDSC may be harmful. Perhaps MDSC that are activated to resolve inflammation blunt cellular responses, enhancing susceptibility to secondary infections [28, 44]. This is consistent with studies that show that impaired T cell function in septic shock is associated with poor outcome [45]. Our sepsis data also concur with data from cancer patients where increased circulating MDSC are associated with more aggressive disease [46].

This study is the first characterisation of diminished T cell zeta-chain expression in human sepsis patients. Downregulation of T cell zeta-chain is a frequent mechanism of immune suppression occurring in patients with chronic hepatitis B, HIV, leprosy, lupus and cancer [30, 47–50] (see also review [51]).

Although some human MDSC have staining profiles consistent with immature granulocytes [28], the neutrophil-MDSC co-purifying with PBMC from sepsis patients appear to be mature granulocytes. In particular, high CD66b, high CD45RO and low CD33 expression are all consistent with mature granulocyte staining [38], and concur with the mature neutrophil phenotype described in renal cell carcinoma patients [52] and in patients with other advanced cancers [31]. The reduced buoyancy of the interphase neutrophils in sepsis patients may represent hyperactivated, degranulated mature granulocytes. Hypodense granulocytes have been described in patients with advanced cancer [31], renal cell carcinoma [30, 52], cutaneous T-cell lymphoma [53], and trauma patients [24]. Pro-inflammatory mediators, including IL-1ß, IL-6 and VEGF can induce MDSC [54, 55], and it was notable that interphase neutrophils correlated with plasma IL-6 concentrations, but not plasma VEGF concentrations in sepsis. Perhaps the inflammatory environment of sepsis may contribute to the accumulation of neutrophil-MDSC. Indeed Schmeilau *et al.* demonstrated that PMN from a healthy donor activated with *N*-formyl-L-methionyl-L-leucyl-L-phenylalanine co-purify with PBMC and suppress T cells in a dose-dependent manner [31]. *In vivo*, neutrophils induced by acute inflammation, following LPS administration to volunteers, fulfil the role of MDSC and suppress T cell proliferation [25].

The role of neutrophils in sepsis has long been controversial with variations in their circulating number (high, low, or with >10% of immature cells) and conflicting reports concerning their functional status [56]. The inability to distinguish PMN and interphase neutrophil-MDSC in automated cell counts may have clouded the interpretation of sepsis neutrophil data. While PMN are beneficial through appropriate responsiveness to chemotactic factors released by infection and bacterial phagocytosis, our data suggest that excessive induction of neutrophil-MDSC will suppress T cell responses by downregulating the zeta-chain compromising cellular responsiveness. Interphase neutrophil-MDSC may represent a mechanism to sequentially downregulate T cell zeta-chain and extinguish effector responses [57] during the transition to memory cells. We speculate that neutrophil-MDSC have a role in modulating T cell responses in sepsis, but excessive numbers and prolonged detection appear to contribute to greater disease severity.

Our initial use of cryopreserved samples precluded direct comparisons between the atypical interphase neutrophils and the PMN. To compare suppressive capacity of the neutrophil-MDSC and matched PMN, additional patients were recruited and comparative assays on fresh samples were done. Suppressive capacity was only detected in the atypical interphase neutrophils. The role of PMN, the triggering of neutrophil-MDSC and the functional differences between the populations deserves further evaluation. Indeed further studies are warranted to determine if interphase neutrophil-MDSC frequency, duration or apoptosis/necrosis are predictors of patient mortality.

Although we did not investigate associations between MDSC number and arginase mRNA, our results suggest that arginase is one mode of action of interphase neutrophil-MDSC suppression in sepsis. Interphase neutrophil-MDSC express arginase and consume L-arginine, and the addition of an arginase inhibitor restores T cell proliferation. Our inability to detect arginase in culture supernant suggests that arginase was active internally in the interphase neutrophils and not liberated in cell culture. These data are consistent with a mechanism of suppression attributable to human MDSC [54], and in particular, mature activated granuloctyes in renal cell carcinoma [52].

## Conclusion

In conclusion, we demonstrate that neutrophils which co-purify with PBMC are MDSC that suppress T cell proliferation by impairing T cell zeta-chain expression. The percentage of interphase neutrophil MDSC in sepsis patients is proportional to disease severity and correlates with plasma IL-6 concentrations. Taken together, these results suggest the inflammatory milieu of sepsis increases circulating interphase neutrophil-MDSC, which suppress T cell function. As in cancer [46], neutrophil-MDSC are associated with more severe disease and may be a major link between inflammation and T cell suppression in sepsis. This study indentifies for the first time neutrophil MDSC as mediators of impaired T cell responses during acute human infection. A clear understanding of the role of interphase neutrophil-MDSC in severe sepsis may help identify new targets for novel adjunctive treatment.

## Key messages

-  Hypodense neutrophils express arginase in sepsis and suppress T cell function by decreasing expression of the T cell zeta-chain.
-  Patients with higher numbers of hypodense neutrophils have more severe disease, higher plasma IL-6 and slower recovery of T cell function.

## Electronic supplementary material

Additional file 1: Table S1: Supplementary primer table. (DOCX 14 KB)

Additional file 2: Figure S1: Cytospin images of total interphase cells and polymorphonuclear neutrophils (PMN) in a representative septic shock patient captured using the Aperio XT at 40× magnification. Arrows indicate interphase neutrophils. (PDF 222 KB)

Additional file 3: Figure S2: Flow cytometric detection of CD16, CD49d, CD62L and CD54 expression on interphase peripheral blood mononuclear cells (PBMC) and interphase neutrophils in two representative septic shock patients. (PDF 84 KB)

Additional file 4: Figure S3: Cryopreserved peripheral blood mononuclear cells (PBMC) were thawed and cell division was evaluated using Ki67 in total interphase cells, the interphase PBMC after depletion of CD66b + interphase neutrophils- and following add-back of the isolated cells. (PDF 29 KB)

## Abbreviations

APACHE: acute physiology and chronic health evaluation
CD: cluster of differentiation
CFSE: carboxyfluorescein diacetate succinimidyl ester
ELISA: enzyme linked immunosorbent assay
HIV: human immunodeficiency virus
HPLC: high pressure liquid chromatography
IDT: Integrated DNA Technologies
IFN: interferon
IL: interleukin
iNOS: inducible nitric oxide synthase
L: ligand
LLD: lower limits of detection
MDSC: myeloid-derived suppressor cells
mRNA: messenger RNA
nor-NOHA: N-Hydroxy-nor-L-arginine, diacetate salt
PBMC: peripheral blood mononuclear cells
PCR: polymerase chain reaction
PMN: polymorphonuclear neutrophils
SIRS: systemic inflammatory response syndrome
SOFA: sequential organ failure assessment
TCR: T cell receptor
TNF: tumour necrosis factor
VEGF: vascular endothelial growth factor.
