# Supplementary material for: Neutrophils with myeloid derived suppressor function deplete arginine and constrain T cell function in septic shock patients
Source: Crit Care. 2014 Aug 1;18(4):R163. doi: 10.1186/cc14003 (PMC4261583; doi:10.1186/cc14003)
Supplement: Supplementary file 1 — Additional file 1: Table S1: Supplementary primer table. (DOCX 14 KB) [file 13054_2014_2719_MOESM1_ESM.docx]

| Gene | Forward Primer | Reverse Primer | Probe # | Probe cat# | Probe sequence |
| --- | --- | --- | --- | --- | --- |
| ARG1 | acatcacagaagaaatctacaaaacag | tgctgtgttcactgttcgagt | 60 | 4688589001 | tggggaag |
| ARG2 | cgggggactaacctatcgag | ttcaacaagatccagtgctga | 73 | 4688961001 | gctgagga |
| GAPDH | agccacatcgctcagacac | gcccaatacgaccaaatcc | 60 | 4688589001 | tggggaag |
| HPRT1 | tgaccttgatttattttgcatacc | cgagcaagacgttcagtcct | 73 | 4688961001 | gctgagga |
