# Supplementary figures and images for: Neutrophils with myeloid derived suppressor function deplete arginine and constrain T cell function in septic shock patients
Source: Crit Care. 2014 Aug 1;18(4):R163. doi: 10.1186/cc14003 (PMC4261583; doi:10.1186/cc14003)

## Interphase

Day 0

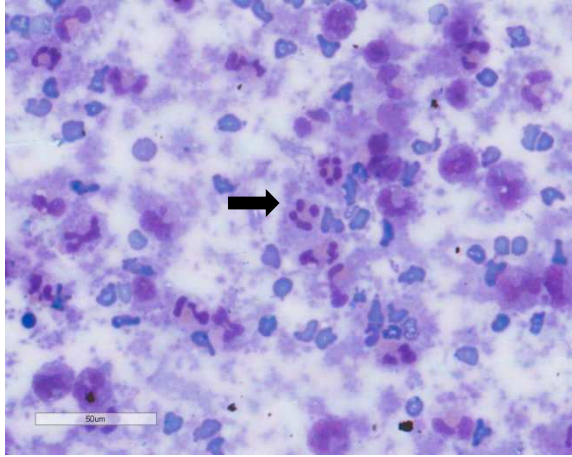

Day 3

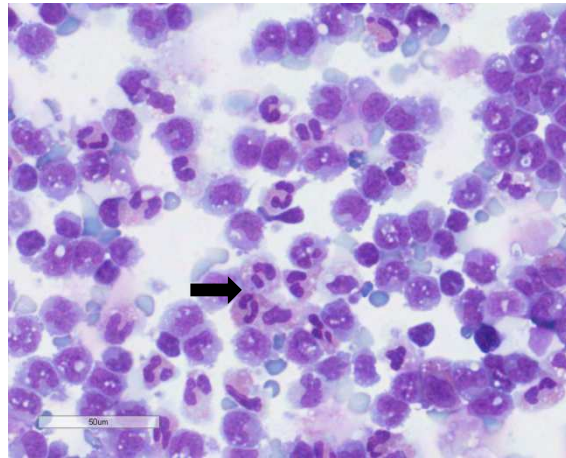

Day 4

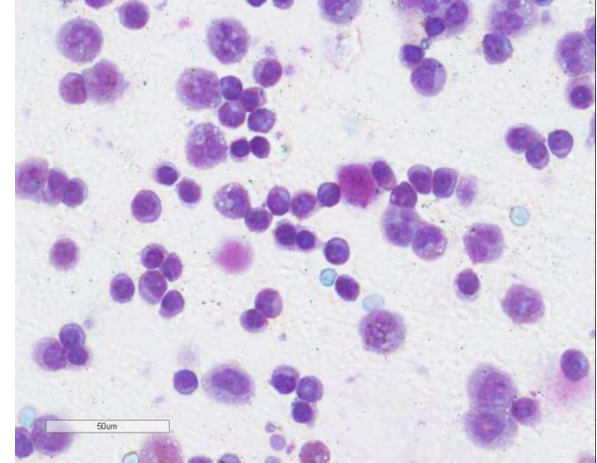

## PMN

Day 0

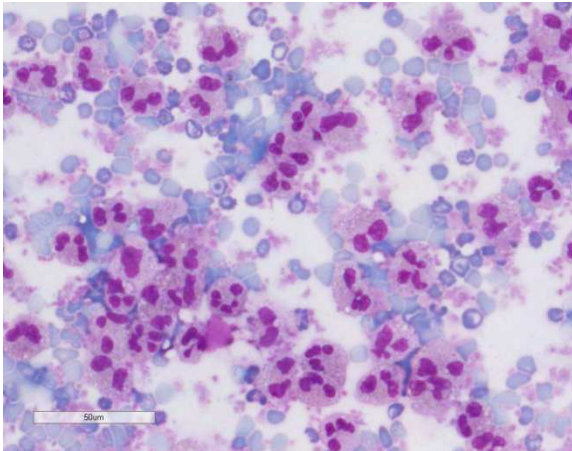

Day 3

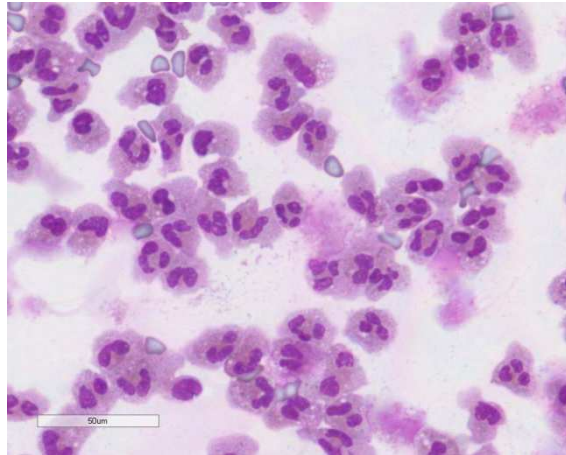

Day 4

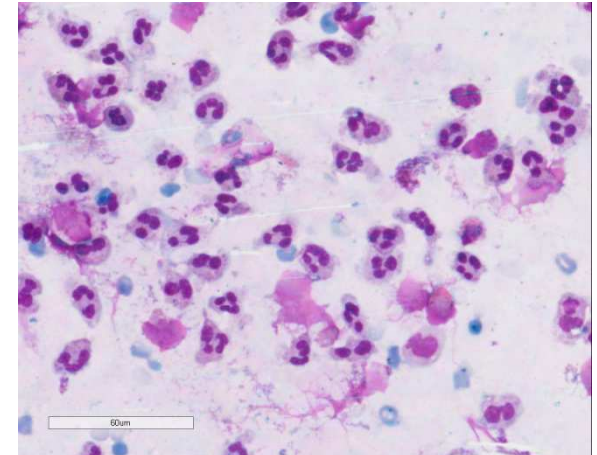

Supplement: Supplementary file 2 — Additional file 2: Figure S1: Cytospin images of total interphase cells and polymorphonuclear neutrophils (PMN) in a representative septic shock patient captured using the Aperio XT at 40× magnification. Arrows indicate interphase neutrophils. (PDF 222 KB) [file 13054_2014_2719_MOESM2_ESM.pdf]

## Person A

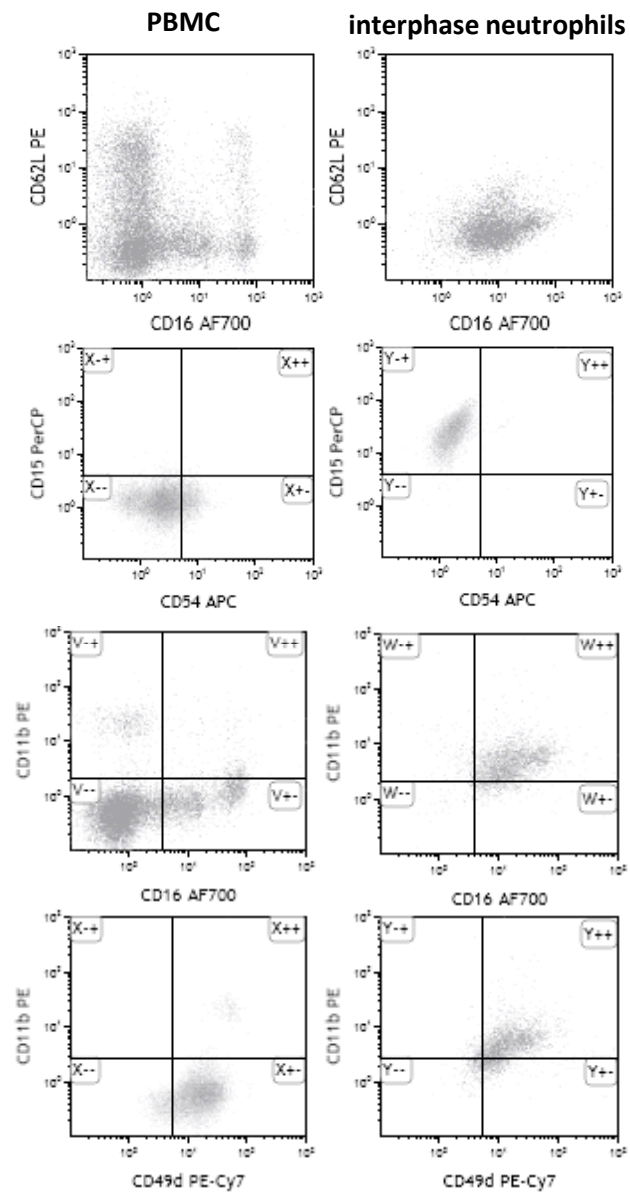

## Person B

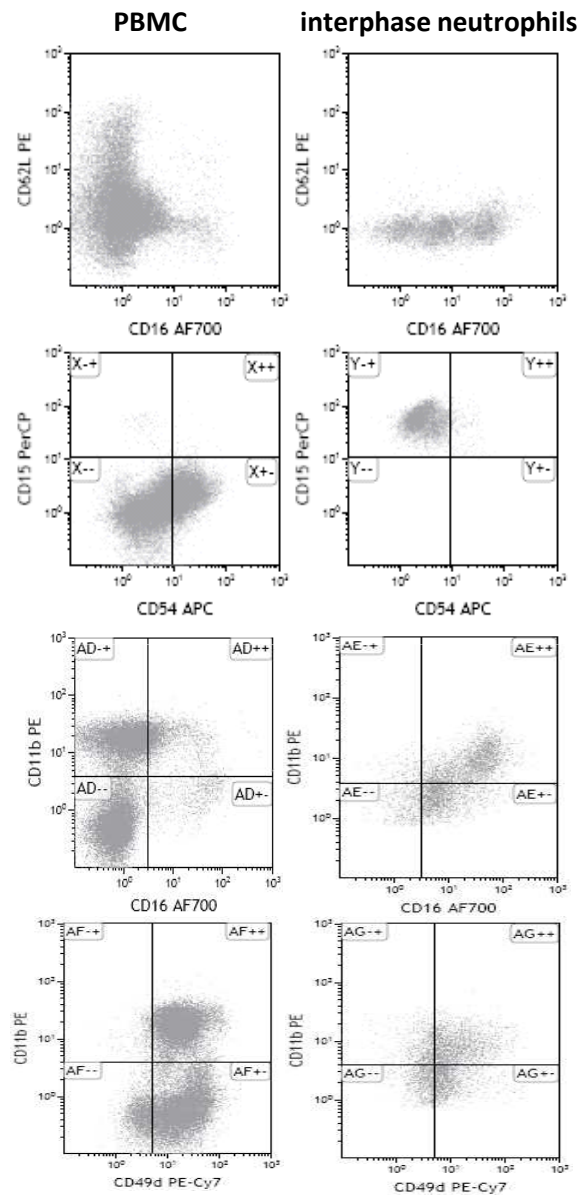

Supplement: Supplementary file 3 — Additional file 3: Figure S2: Flow cytometric detection of CD16, CD49d, CD62L and CD54 expression on interphase peripheral blood mononuclear cells (PBMC) and interphase neutrophils in two representative septic shock patients. (PDF 84 KB) [file 13054_2014_2719_MOESM3_ESM.pdf]
