# Supplementary material for: Neutrophils with myeloid derived suppressor function deplete arginine and constrain T cell function in septic shock patients
Source: Crit Care. 2014 Aug 1;18(4):R163. doi: 10.1186/cc14003 (PMC4261583; doi:10.1186/cc14003)
Supplement: Supplementary file 4 — Additional file 4: Figure S3: Cryopreserved peripheral blood mononuclear cells (PBMC) were thawed and cell division was evaluated using Ki67 in total interphase cells, the interphase PBMC after depletion of CD66b + interphase neutrophils- and following add-back of the isolated cells. (PDF 29 KB) [file 13054_2014_2719_MOESM4_ESM.pdf]

unstimulated **Stimulated** **Plots show a minimum of 50,000 CD3+ T cells**

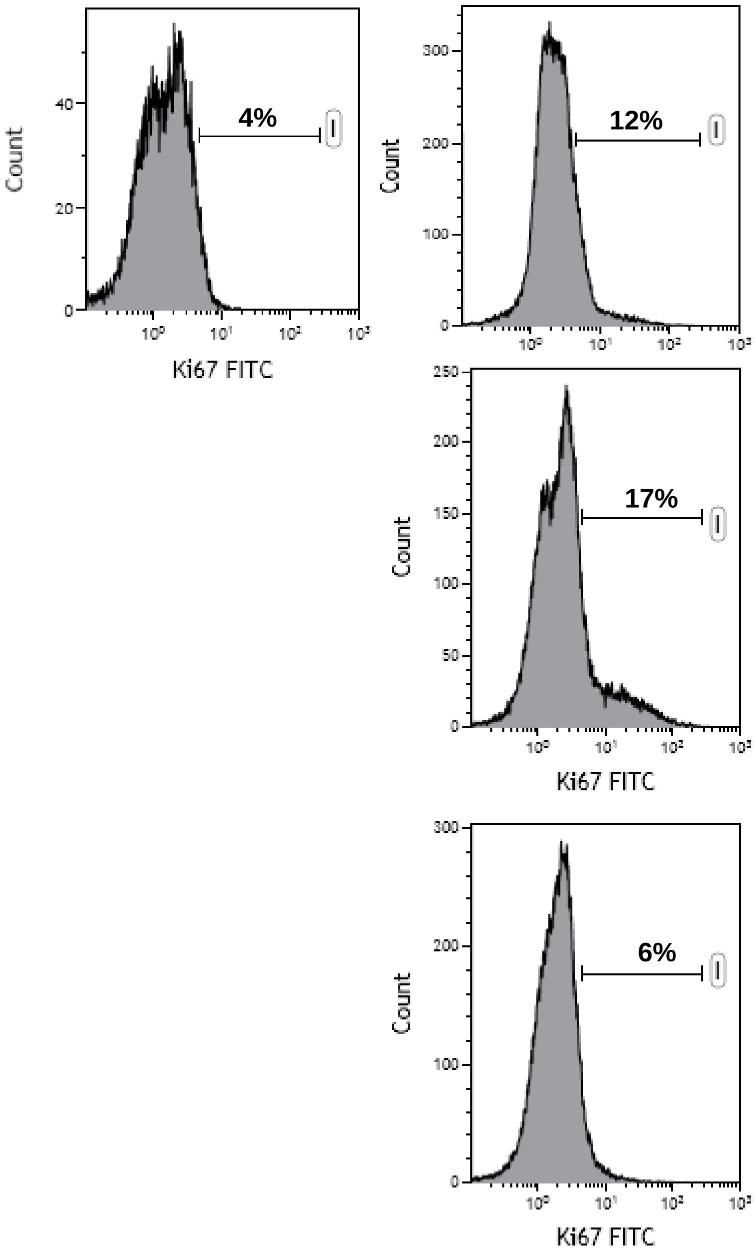

Total interphase cells
PBMC and neutrophil-MDSC

PBMC (interphase depleted of CD66b+ cells)

PBMC plus CD66b+ cell add back
